# Characterization of the complete chloroplast genome of traditional Tibetan herb, *Rheum Pumilum* Maxim. (Polygonaceae)

**DOI:** 10.1080/23802359.2019.1698344

**Published:** 2019-12-13

**Authors:** Rui Li, Xiaofeng Zhang, Jiuli Wang, Dangwei Zhou, Huan Wang, Shengbo Shi, Tingfeng Cheng

**Affiliations:** a College of Medicine, Xi’an International University, Shaanxi, P. R. China;; b Key Laboratory of Adaptation and Evolution of Plateau Biota (AEPB), Northwest Institute of Plateau Biology, Chinese Academy of Sciences, Qinghai, P. R. China;; c College of Ecological Environment and Resources, Qinghai Nationalities University, Xining, China

**Keywords:** *Rheum pumilum* Maxim., chloroplast genome, phylogenetic analysis

## Abstract

The complete chloroplast genome sequence of traditional Tibetan herb, *Rheum pumilum* Maxim. was analyzed. The complete chloroplast genome of *R. pumilum* is 162,213 bp in size and has a 27.27% GC content. In the typical circular quadripartite structure, there was a pair of inverted repeat (IR) regions with 31,023 bp in length, which separated by a large single-copy (LSC) region (87,424 bp) and a small single-copy (SSC) region (12,743 bp). The chloroplast genome of *R. pumilum* contained 131 unigenes, which was composed of 86 protein-coding genes, 37 tRNA and 8 rRNA genes. Moreover, 238 SSRs were identified and 58.8% of them existed in LSC region. A maximum likelihood (ML) phylogenetic analysis based on chloroplast genomes indicated that *R. pumilum* was closely related to *R. pulmatum*, *R. tangutica*, and *R. officinale*. Our results would provide a valuable resource for resource utilization and the phylogenetic studies of Rheum in Polygonaceae.

*Rheum pumilum* Maxim. (XiaoDahuang in Chinese) is an alpine perennial herb, belongs to Polygnonaceae family. Unlike *Rheum palmatum*, *Rheum tanguticum*, and *Rheum officinale*, its leaves appear ovule or wide ovule and shoot is dwarf (5–25 cm). This species distributes in sand soil, alpine meadow or shrub from 3000 m to 4700 m on the Qinghai-Tibet Plateau (Liu 1996), and the stout roots also have great medicinal values (Wang et al. 1988; Zhang 2004; Dong et al. 2016). Although complete chloroplast (cp) genomes of *R. palmatum*, *R. tanguticum*, and *R. officinale* have been studied (Fan et al. 2016; Zhou et al. 2018), there is no record about this species till now. In this paper, we sequenced and assembled the complete chloroplast of *R. pumilum* using Illumina Hiseq platform. The cp genome was annotated and submitted to the Genbank with the accession number (MN652917).

We collected fresh leaves from a single individual of this species from Daban Mountain (N37.34°, E101.40°; Alt. 3940 m), Menyuan County, Qinghai, China, and dried leaves with silica gel. Voucher speciements were deposited in the herbarium of Northwest Institute of Plateau Biology, CAS (HNWP, Zhou2019021). Total DNA was extracted from the fresh leaves with the DNeasy Plant MiniKit (QIAGEN, CA, USA) according to the manufacturer’s instructions. DNA quality was assessed based on spectrophotometry and electrophoresis in 1% (w/v) agarose gel, and then the good integrity and purity DNA was used for library construction and sequencing with the Illumina Hiseq platform (San Diego CA, USA) at Genepioneer Biotechnologies Inc., Nanjing, China.

In total, we obtained about 21,341,719 high quality clean reads. The cp genome was assembly using NovoPlasty software (Dierckxsens et al. 2017) and the previously published cp genome of *R. palmatum* (Fan et al. 2016) was used as seed reference. We visualized the genome by Geneious version8.05 (Kearse et al. 2012). Gene anonation firstly perform with DOGMA (Wyman et al. 2004) and CpGAVAS (Liu et al. 2012), then corrected manually with the Geneious (Kearse et al. 2012). Finally, the physical map of cp genome of *R. pumilum* was done with CpGAVAS (Liu et al. 2012). The complete cp genome sequence and its annotations were submitted to Genbank (MN652917).

The complete chloroplast genome of *R. pumilum* is 162,213 bp in size and has a 27.27% GC content. In the typical circular quadripartite structure, there was a pair of inverted repeat (IR) regions with 31,023 bp in length, which separated by a large single-copy (LSC) region (87,424 bp) and a small single-copy (SSC) region (12,743 bp).The chloroplast genome of *R. pumilum* contained 131 unigenes, which was composed of 86 protein-coding genes, 37 tRNA and 8 rRNA genes. Moreover, 238 SSRs were identified and 58.8% of them existed in LSC region. Compared the four cp gnome of Polygonaceae, most genes were conserved and the variation displayed in the intergeneric regions.

To identified the phylogentic relationship of *R. pumilum* and other 24 species, 65 protein coding genes extracted from the chloroplast genome of 16 taxa, which including 6 species of Polygnonaceae and species of *Amborella trichopoda* in Amborellacea was set as the outgroup. Sequences were aligned using the program MAFFT (Katoh and Standley 2013), and maximum likelihood (ML) analysis was performed by RaxML based on Kimura 2-parameter model with 1000 bootstrap replicates (Alexandros et al. 2017). The phylogenetic tree displayed that *R. pumilum* were closer clustered with *R. palmatum*, *R. tanguticum*, and *R. officinale* while *R. witrochii* is out of them clearly (Figure 1). Our cp genome data of *R. pumilum* would facilitate population, genetic identification and cp genetic engineering research of this traditional Tibetan herb in the future.

**Figure 1.**
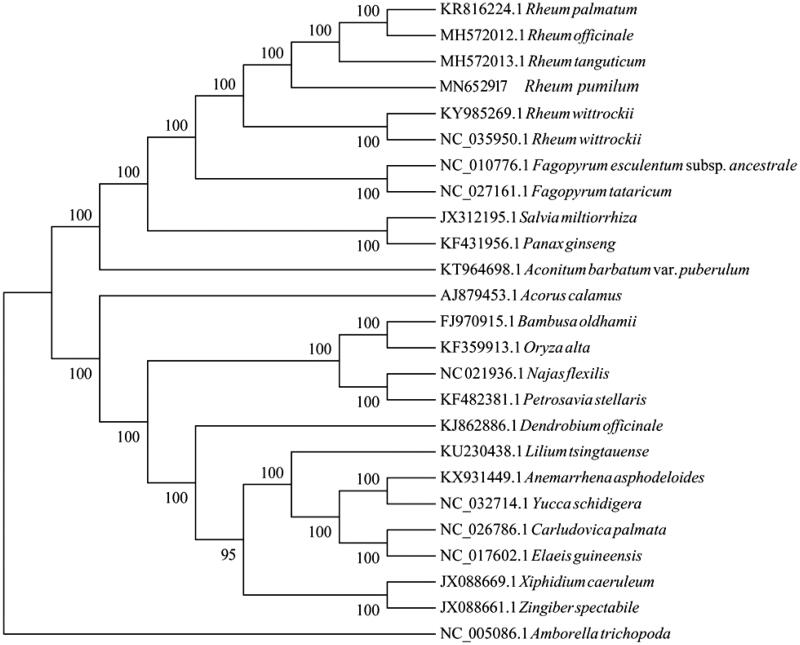
Maximum likelihood (bootstrap repeat is 1000) phylogenetic trees of *R. pumilum* A. and related species based on the whole cp genomes. Chloroplast genomes: *Rheum palmatum* (KR816224.1), *Rheum officinale* (MH572012.1), *Rheum tanguticum* (MH572013.1), *Rheum pumilum* (MN652917, this study), *Rheum wittrockii (*KY985269.1), *Rheum wittrockii* (NC_035950.1), *Fagopyrum esculentum* subsp.ancestrale (NC_010776.1), *Fagogyrum tataricum* (NC_027161.1), *Salvia miltiorrhiza* (JX312195.1), *Panax ginseng* (KF431956.1), *Aconitum barbatum* var.puberulum (KT964698.1), *Acorus calamus* (AJ879453.1), *Bambusa oldhamii* (FJ970915.1), *Oryza alta* (KF359913.1), *Najas flexilis* (NC021936.1), *Petrosavia stellaris* (KF482381.1), *Dendrobium officinale* (KJ862886.1), *Lilium tsingtauense* (KU230438.1), *Anemarrhena asphodeloides* (KX931449.1), *Yucca schidigera* (NC_032714.1), *Carludovica palmate* (NC_026786.1), *Elaceis guineensis* (NC_017602.1), *Xiphidium caeruleum* (JX088669.1), *Zingiber spectbile* (JX088661.1), *Amborella trichopoda* (NC_005086.1).
